# Partial Thyroidectomy in the Treatment of Exophthalmic Goitre

**Published:** 1908-02

**Authors:** Aime Paul Heineck

**Affiliations:** Chicago, Adjunct Professor of Surgery, University of Illinois; Surgeon to Cook County Hospital, etc.


### TEXAS MEDICAL JOURNAL.

Established July, 1885.

F. E. DANIEL, M. D., - - - - Editor, Publisher and Proprietor

PUBLISHED MONTHLY.—SUBSCRIPTION $1.00 A YEAR.

VOL. XXIII. AUSTIN, FEBRUARY, 1908. No. 8.

The publisher is not responsible for the views of contributors.

For the Texas Medical Journal.

### Partial Thyroidectomy in the Treatment of Exophthalmic Goitre.

**(a) Its Basis, (b) Its Results, (c) Its Technique.**

BY AIME PAUL HEINECK, CHICAGO,
Adjunct Professor of Surgery, University of Illinois; Surgeon to Cook County Hospital, etc.

So as to convey a definite conception of what we understand and of what we mean by the term exophthalmic goitre, at the outset we will quote two definitions, one from a popular text-book of practical medicine, and the other from an equally authoritative text-book of practical surgery.

(a) Exophthalmic goitre is an affection, the chief symptoms of which are goitre, exophthalmos, tachycardia and tremor. In pronounced cases, other symptoms, chiefly of a nervous nature, are present.[10]

(b) Exophthalmic goitre is a disease characterized by an enlargement of the thyroid gland, palpitation and increased frequency of the heart's action by proptosis, fine tremor and general nervousness. By goitre we understand an enlargement of the thyroid gland, involving the entire structure, which may be either partial or symmetrical, as nodules and cysts. By tachycardia, we wish to denote a disturbance in the action of the heart, which is expressed in increased frequency. George P. Murray (*Lancet,* London, 1905, p. 1370, Vol. 11) noticed an enlargement of the thyroid body in 172 out of 180 cases. In five of the remaining cases, there had been a goitre at an earlier stage of the disease. The enlargement of the gland, according to the same author, is generally uniform. The latter statement is contradicted by equally good observers. It is not in accord with my experience. It is important to remember that any individual symptom of exophthalmic goitre may be inconspicuous, may be absent during a part or the entire course of any individual case of the disease. We must also remember that those symptoms vary in relative degree in different cases. According to some authors, exophthalmos is absent in about one-fourth of the cases. Tachycardia, not of a paroxysmal or of a transitory nature, but of a permanent character, is the most constant symptom of this affection.

Kocher (Berne, Switzerland) says that he has never seen a well-developed case without a goitre. Mayo (Rochester, Minn.), says that in those cases in which there is an apparent absence of goitre, a careful examination will usually disclose a small unilateral or bilateral tumor, which lies deeper but is firmer than normal thyroid tissue. The tremor in this condition is rapid and vibratory, there being as many as eight or ten vibrations per second.

There are primary and secondary forms of this affection. From an etiological, pathological and therapeutical standpoint, the classification into primary and secondary forms is consistent, is valuable. In the primary form, there is a concurrent development of the goitre and of the other symptoms characteristic of this affection. In the secondary cases, the symptom-complex of this affection is grafted upon a pre-existing enlargement of the thyroid body. All varieties of goitre, from simple cysts to malignant tumors, irrespective of size, type or stage, may be associated with the symptoms-complex of exophthalmic goitre, and it is further to be noted that in all of the secondary forms, excepting those associated with malignant disease, the prognosis of operative interference is better, both as to early and as to late recovery. (Dean Lewis.)

In our perusal of the literature, the frequency with which diagnostic errors are made concerning this affection, surprised us. It is often thought to exist when absent. Pulsating exophthalmos, a totally distinct malady, seems to have been the most frequent source of error. Chlorosis, also, has not been an uncommon source of error. Many operators do not seem to have differentiated between operations for ordinary goitre and those for exophthalmic goitre (primary or secondary). In a discussion on treatment, these two conditions must always be considered separately.

Authors are not in accord as to the etiology and as to the pathological cause of exophthalmic goitre and the treatment most appropriate for this affection is still a matter of discussion. Owing to the fact that the ultimate cause of this affection is still a matter of *speculation,* theories have been advanced, and have been made use of and are still used as foundations for apparently appropriate lines of treatment. Many of these theories have been abandoned. They were based on insufficient knowledge; on misinterpreted clinical and pathological data.

We will briefly discuss some of the theories which still have advocates: (a) The cardiac theory, (b) compression theory, (c) sympathetic theory, (d) nervous theory, (e) parathyroid theory, (f) thymus theory, (g) the thyroid theory, and then adopt as a working basis that theory which is least in conflict with facts.

Tachycardia, palpitation, thrills, murmurs, displacements, diffusiveness of the apex-beat, increased area of cardiac dullness, due to dilatation or hypertrophy and other symptoms of a cardiac nature, lead many of the early observers, such as Parry, Stokes, Graves, Buton, Beau, to think that the cause of this affection originates or resides in the heart. The cardiac theory is defective, because:

1. Those cardiac disturbances that occur in the presence of exophthalmic goitre can, and frequently do, occur independently of this disease, viz., toxic palpitation and essential tachycardia.

2. They can occur as symptoms in conditions totally distinct from exophthalmic goitre.

3. Cases of exophthalmic goitre occur in which they are absent.

4. The cardiac disturbances met in this affection are not exciting factors. They are due to co-existing or to complicating functional or organic diseases of the musculature or of the various valves of the heart. Long continued, excessive rapid action of the heart may beget organic changes in this organ.

5. In by far the largest number of cases of exophthalmic goitre that have come to the autopsy table, an absence of cardiac lesions has been noted. In some cases moderate hypertrophy with or without ventricular dilatation and endocardial disease was present. The dilatation of the heart has been ascribed to its quick action; the systole is too short to be complete; residual blood gradually entails overdistension and dilatation. This disease can occur in individuals with pathologic hearts, and then the symptoms of the original lesion will be superadded to the cardiac manifestations of the affection we are now discussing.

6. The cure of exophthalmic goitre does not cure any co-existing organic cardiac lesion. Kocher reports two cases of exophthalmic goitre, co-existing with mitral insufficiency. The symptoms of exophthalmic goitre were cured by partial thyroidectomy. The symptoms of mitral insufficiency were uninfluenced and persisted. This shows the non-interdependence of the two conditions.

THE COMPRESSION THEORY.

When Tillaux performed his first thyroidectomy for the relief of exophthalmic goitre, he believed that the affection was caused by the compression exerted by the hypertrophied thyroid gland on the important vascular and nervous structures at the base of the neck. His patient recovered from the operation and from the disease, but the explanation which he advanced was erroneous. He later abandoned the compression theory. It has had many and still has a few eminent advocates. It is untenable, because:

1. It is hard to understand how unilateral compression (the thyroid enlargement may be unilateral though it is more frequently bilateral) can cause bilateral exophthalmos.

2. How unilateral enlargement of the thyroid body can cause unilateral exophthalmos of the opposite side of the body, as in the cases reported Panas and by Gros, etc.[31]

3. Why, if compression be the cause, do the symptoms bear no relation to the degree of thyroid enlargement? It is an acknowledged fact that, in some cases of great severity, it takes the most precise and painstaking palpation to detect any enlargement of the thyroid gland. Easily detectable enlargement of the thyroid gland like every other cardinal symptom of this affection may be absent in individual cases. As a rule, the enlargement of the thyroid is not very great in this disease. The severity of the toxemia in Graves' disease bears no relation to the degree of hypertrophy.

4. Why, if the enlarged thyroid gland compressed any of the neighboring nerves, the recurrent laryngeal nerves, which by virtue of their anatomical location would be the ones most frequently involved, are so infrequently in this affection the seat of compression paralysis? They are far less frequently involved than in cases of simple goitre. There are, at the most, only ten cases of exophthalmic goitre in which paralysis of one or both recurrent laryngeal nerves has been observed.

5. Why massive tumors of the base of the neck do not determine exophthalmic symptoms? Why large parenchymatous or cystic goitres can exist for years and never be productive of, or be associated with, the symptom-complex of this disease. If compression were a determining factor of this disease, its action would be invariable. Under like conditions, it would produce like results.

6. If the symptoms were due to pressure, they would immediately and completely disappear on the removal of the compressing agent. We know that they do not.

### THE SYMPATHETIC THEORY.

This theory was advanced by Kobens, defended by Trousseau, Oppenheimer and others. Disturbances of the sympathetic system with or without structural change can explain some of the symptoms of this disease. They fail to explain many. Observers of note, such as Abadie, Jaboulay, Poncet, Jonnesco, etc., have looked upon the cervical sympathetic trunk and its ganglia as the primary seat of the disease, as the *fons et origo mali.* Some have advised, and the latter three had devised and performed operations upon the sympathetic nerves for the relief of primary exophthalmic goitre. Jaboulay, in some cases of primary exophthalmic goitre, performs a bilateral division of the cervical sympathetic nerve trunk. In other cases of the primary form of this disease he performs a bilateral resection of both nerve trunks. Jonnesco performs a complete resection of both cervical sympathetic trunks, including the upper, the middle and the lower cervical ganglia. The value of these operative procedures has been much discussed. The uniformly good results obtained by their originators have not been obtained, have not been approached, by other equally dextrous and competent operators. Operations on the sympathetic for the relief of this condition have fallen almost into complete disuse.

Boissou (*Thèse de Paris*) collected all the cases of exophthalmic goitre that he could obtain up to 1898, for the relief of which an operation on the sympathetic nerve or ganglia had been performed. He collected 27 cases and analyzed 23, as the other four, for one reason or another, prove nothing. In these cases there were 3 complete cures, 8 marked improvements, 3 deaths. His conclusions are: "Successes are rare. In cases benefited, it is sometimes one symptom, sometimes another, which is improved; and the improvement is sometimes immediate, sometimes late, sometimes there are relapses in the improvement. Neither by division or excision, total or partial, have more brilliant results been obtained. From the physiological point of view, all is chaos. There is no relation between the phenomena noted by the surgeons and those observed by the physiologists, also the surgical effects are in discord with one another." Schiff, in his experiments, noted that no effects on thyroid gland followed the section of the sympathetic cervical nerves. The sympathetic theory is now only held by a few observers.

In a certain number of cases lesions of the sympathetic nerve have been reported. The changes were such as are often found in many other diseases; such as are often found in health. Abadie, one of the most ardent advocates of the sympathetic theory, says: "The nervous sympathetic trunk is neither diseased or degenerated; its tissue is intact and does not show any lesion." However, he gives no detailed report of observation. Hale White[1] has shown by a careful series of investigations on patients dying from other causes that variations in the size and in the vascularity of the cervical sympathetic ganglia, as observed by the naked eye, have no significance; that the cellular elements are found in such various degrees of number and integrity, and that the fibrous stroma is so variable as to prevent any definite statement. He further says: "This evidence (his observations and those of others) seems to me conclusively to show that the lesion is not in the sympathetic nerves."

The only recent observer that has found positive changes in the sympathetic cervical ganglia is Greenfield.[3] The changes which he noted are not characteristic. In two cases examined, he found these ganglia to be swollen, markedly hyperemic and infiltrated with leucocytes. Degenerative changes in the ganglion cells were present. Ehrich is of the opinion that what degenerative changes have been found in the sympathetic nerves are purely secondary. The only changes which he found were vacuolation and fat droplets. (*Beitrage zur klinischen Chirurgie*, 1900, Vol. XXVIII, p. 136.)

In Temoin's case (*Revue de Chir.*, Vol. 18, 1898, p. 10) microscopical examination of the sympathetic cervical ganglion showed nothing definite. Numerous observers of great competence and acuity of observation report negative findings. Achard and Joffroy examined the cervical sympathetic ganglion in four cases of exophthalmic goitre and found it absolutely normal. Ranvier examined one case; his findings were the same. The observations of Marie and Marinesco,[4] of Mendel,[5] of Joffroy and Achard,[6] taken in connection with those of Wilks, of Barth, and of Déjerine, suffice to convince one that exophthalmic goitre is not due to an organic disease of the sympathetic nervous system.

If we assumed that this disease is dependent on a functional involvement of the cervical sympathetic trunk and its ganglia, we could not satisfactorily explain why in the same nerve trunk there is a stimulation of some fibres, depression of others and functional integrity of the remaining fibres. Dilatation of the pupil, which is perhaps the most constant symptom of stimulation of the cervical sympathetic nerve, is notably absent in Graves' disease. The fact that there are no invariable ocular of pupillary symptoms or signs, further argues against the primary involvement of the sympathetic as being the cause.

THE NERVOUS THEORY.

The nervous theory is supported by Sattler, Putnam, Mendel, etc. Owing to the apparent sudden onset of exophthalmic goitre, to its frequent occurrence in individuals of the same families, be they ascendants, descendants or collaterals; owing to its far more frequent occurrence in women, and to the fact that the sufferers of this affection frequently belong to neuropathic families, in which cases of epilepsy, hysteria, chorea, or even some form of insanity have occurred; owing to its frequent association with nervous affections of a functional or of an organic nature, such as hysteria, neurasthenia, tabes dorsalis, syringo-myelia, epilepsy, etc., and also to the many nervous manifestations of this disease, such as tremor, general nervousness, mental disturbances, paresis and paralysis, etc., many clinicians have been led to believe that this affection is dependent upon either a functional or an organic disease of the cerebro-spinal nervous system. If we consider this affection a neurosis; that is, a nervous disease having no demonstrated organic basis, we will have to classify it among such functional nervous diseases as hysteria, chorea, neurasthenia, etc. We can not so consider it, as we know that this disease is always associated with definite histo-anatomical changes in an organ not belonging to the nervous system. As to its being a nervous disease with appreciable anatomical lesions in the nervous system, we will submit the evidence for and against this contention.

Edmunds, W. (London, *Lancet,* 1901, Vol. 1, p. 1317), and other investigators have found that the nerve lesions of exophthalmic goitre are extremely uncertain. One observer has found one change; one, another. And when in a case of Graves' disease, a systematic examination of the nervous system has been made, the findings have usually been negative. The lesions that have been found vary considerably in the different cases, though each one may be definite enough in itself. The lesions are constant; are not characteristic. We acknowledge that many of the symptoms clearly show that the normal functions of the nervous system are deranged, but similar disturbances occur in other toxemic states, and, in the absence of organic changes in any part of the nervous system, they can not be adduced as evidence of a nervous cause of the disease. No constant change in the peripheral nervous system has been noted. Mueller and several other observers have occasionally found changes in the pneumogastric and recurrent nerves; a few degenerate fibres being present in these nerves. As a rule, however, these nerves have been found normal. Mueller decided that the degenerative changes in the vagus nerve were secondary. Sir Victor Horsley noted in his experiments that division of the recurrent laryngeal nerves was without effect upon the thyroid gland.

In the spinal cord, in those cases where the disease has co-existed with organic nervous diseases such as tabes dorsalis, at the post-mortem table, there will be found the anatomical changes characteristic of that organic disease. In cases of exophthalmic goitre not complicated by an organic spinal cord disease, there is noted a total absence of demonstrable anatomical changes. Greenfield believes that he noted in some cases that came to autopsy changes similar in nature, but less marked in degree, to those which are noted in tetanus and hydrophobia. Most observers, however, have noted no change other than slight degrees of congestion, and even this slight degree of congestion was present in only a few instances.

As to the brain, Eger noted in one case adhesions of the dura mater over the convexity. Peter, in another case, found a large hemorrhagic focus in the pons and medulla. Vandervelder and Le Boeuf report having found a vascular neuro sarcoma beneath the gray matter of the left superior parietal convolution. In a single case, Johnson and Mueller each found hyperemic softening of the convolutions. We must bear in mind that these findings are isolated findings; they are exceptional occurrences; they are coincidences. In most of the cases that have come to the autopsy table, the brain and its membranes were not the seat of changes detectable with our present methods of examination.

Filehne, in experimenting on rabbits, found that when he transacted the restiform bodies at different levels that he was able to produce tachycardia, exophthalmos, and, occasionally, the autopsies showed swelling of the thyroid gland. Bienfait partially confirmed these results in 37 per cent of his cases.

Tedeschi, as a result of experiments personally conducted, came to the following conclusions:

1. That injury to the restiform bodies in rabbits produces artificially exophthalmic goitre.

2. In animals thus affected, when the symptoms have disappeared, they can be reawakened in part by hyperthyroidization.

3. In animals in whom the thyroid body has been removed, lesions of the restiform bodies do not produce exophthalmic goitre.

4. In animals in whom the exophthalmic goitre symptom-complex has been produced by loss of the restiform bodies, removal of the thyroid gland diminishes or completely banishes the greater part of the symptoms.

The objections to considering atrophy or degenerative changes in the restiform body as being the cause of the disease under discussion are many. Atrophy of the restiform bodies has been found in two cases of tabes dorsalis, in which symptoms of exophthalmic goitre were not present. (Oppenheimer.[2]) Lesions of the restiform bodies would not explain the partial paraplegias, would not explain the muscular atrophy, the absent or diminished patellar reflex, etc. Joffroy and Achard (*Arch. de Med. Experimentale,* 1893, Vol. 6, p. 807) were unable in their experiments on animals to reproduce the symptoms of Graves' disease by provoking injuries of the bulb. "That lesions of the restiform bodies are quite exceptional is evidenced by the fact that in 16 cases reported by recent observers (Joffroy, Siemerling, Koeppen, Goldscheider, Mueller, Achard) the restiform bodies are described as absolutely normal. (Francis P. Kinnicult)."

We see that by all that precedes that exophthalmic goitre is not due to a constant and characteristic nervous lesion, demonstrable in every case. Post-mortem observations teach us that in most cases of this disease there is a total absence of anatomical lesions of the cerebro-spinal and sympathetic nervous systems; that is, of such lesions as can be revealed by our present methods of examination.

THE PARATHYROID THEORY.

Not much is known concerning these bodies. A great deal of speculation concerning the function of these bodies has been indulged in. They are four in number, two on each side. They contain no follicles, no colloid substance and are composed of epithelial cells. It has been suggested by some (Edmunds, MacCallum) that exophthalmic goitre might be dependent upon functional or structural disturbances of one or of more of these bodies, or upon their absence. We do not as yet adopt this theory as a working basis, because:

1. Our knowledge concerning the physiology and the pathology of these glands is as yet too limited.

2. Parathyroid therapy is useless in this affection. Walsh[15] came to the conclusion that there are no grounds for the idea that insufficiency of the parathyroid plays an important part in Graves' disease.

3. The changes that have been found in the parathyroid bodies have been noted by Walsh, Humphrey and Berkeley after death from other diseases.

4. In nine cases of exophthalmic goitre in which the parathyroid bodies were examined microscopically by MacCallum[16] they were found practically normal, there being at most, in a few of the cases, only a slight diminution in size and an increase in the fibrous stroma.

Shattuck found nothing abormal in the parathyroids from exophthalmic goitre patients which he examined microscopically. Benjamins[17] examined the glandulæ parathyroidæ in sixteen simple and three Basedow's goitres without finding any noteworthy change.

### THE THYMUS THEORY.

This theory, as an explanation of exophthalmic goitre, was suggested by the following facts:

1. In some of the cases of exophthalmic goitre that have come to the autopsy table, this organ was hypertrophied. Dinkler, Joffroy, Soupault, Mobia, Spencer, Marie and other authors have commented upon this marked hypertrophy. It is most always associated with great vascularity of the organ.
2. Some cases have apparently been benefited by the use of thymus gland or of some of its preparations.

Our non-acceptance of this theory is based upon the following facts:

1. Our knowledge of the physiology and of the pathology of the thymus gland is as yet too limited. Very little is known concerning the functions of this organ.
2. Thymus therapy in the opinion of by far the greater number of clinicians is useless in the treatment of exophthalmic goitre.
3. Exophthalmic goitre occurs in the absence of hypertrophy of the thymus.
4. Hypertrophy of the thymus occurs in the absence of exophthalmic goitre.
5. The thyroid theory offers a far better working theory.

Mackenzie states that he treated 20 cases of this disease with thymus extract, and compared them with 20 similar cases treated without thymus, but could see no decided difference in the results obtained.

### THE THYROID THEORY.

Though it has not been demonstrated beyond scientific contradiction, that the thyroid gland is the sole primary cause of the disease, we are believers in the thyroid theory, because:

1. There is present some structural alteration of the thyroid body in all cases of exophthalmic goitre. This applies to the secondary as well as to the primary cases. Most recent observers have come to the conclusion that the histology of the thyroid gland in Graves' disease is in many respects specific.[19] We must not conclude that because we can not detect any enlargement that, therefore, the gland is not diseased; it always is.[20]
2. Exophthalmic goitre is the direct opposite of myxedema in symptomatology, and pathology and in therapeutical indications.
3. Because the symptoms-complex of this affection can to a certain degree be determined by the ingestion of thyroid gland substance or of its various preparations.
4. Because all those medicinal measures which tend to decrease the functional activity of the gland also tend to lessen the severity of the symptoms.
5. Because in the cases which we have collected and which we report recovery from the disease, in rapidity and in completeness, has been in proportion to the extent of the gland-tissue removed short of its entirety.

6. In those cases where the symptoms recurred there was an associated hypertrophy of the remaining portion of the gland. Recovery could be secured by a secondary operation.

7. Because the symptom-complex of this affection finds its most satisfactory explanation by considering the condition a general toxemia, the result of quantitative or qualitative changes, or both, in the secretion of the thyroid gland. The tachycardia, the mental changes, the sweating, the prostration, the increase of body temperature, the diarrhea, are all symptoms that we find in other intoxications.

The anatomical changes noted in the primary and secondary forms of this disease are unlike; so unlike that they, of themselves, make imperative the classification of the disease into primary and secondary forms. In the secondary cases, we agree with Dean Lewis[5] when he says that the goitre in the secondary forms does not differ in structure from the simple or parenchymatous or other goitre, upon which the Basedow's symptom-complex were grafted. Exophthalmic goitre has been observed in simple goitre, in fetal adenomata, in cysts, in carcinoma of the thyroid gland. (Bloodgood, Ehrhardt.) It has been thought that small tumors of the thyroid glands, the seat of secondary Graves' disease, act as irritants, causing an over-activity of the gland, much as a foreign body in the eye will produce an excessive secretion of tears, and the removal of this source of irritation by operation has been followed by a complete relief of the symptoms.

In the primary form of Graves' disease, pathological changes are constantly present in the thyroid gland. Kocher and Reinbach, Brissaud and Langhans have denied the above statement. But the existence of these changes has been confirmed by so many competent observers that their occurrence can no longer be contested. (Greenfield,[10] Askanazy, Haemig, Aubarsch, MacCallum[22] and Ehrhardt.) In 28 primary cases of exophthalmic goitre, MacCallum found the thyroid gland involved in all, although it was not equally involved in every case. It is well to bear in mind that changes may occur in one portion of a gland, and be absent in another. Dean Lewis examined carefully the thyroid gland in four cases of primary exophthalmic goitre. His findings agree practically with those of Greenfield, MacCallum, Edmunds. What are these changes which we considered as constant and almost as characteristic?

1. *Changes in the Follicles Which Are Increased in Number and Which Are Also Changed in Size and Form.*—Instead of appearing round or square in section, they become branded and stellate. Dr. Rodocanachy[23] noted an increase in the number of the alveoli, proliferation of the epithelium and changes in its character. Dean Lewis says: "It seems as if the proliferating epithelium following the lines of least resistance had grown into the follicle; the connective tissue of the follicle is also invaginated so that in any sections the invaginated epithelium with its connective tissue stalk, resembles an intestinal villus." In other parts of the gland, the follicles are unusually small. Many of the follicles contain desquamated epithelial cells. The secreting area of the vesicles is increased by ingrowths from their walls.

2. *Changes in the Character of the Epithelial Cells.*—The cells are changed from the cuboidal to the cylindrical, columnar type. The epithelial proliferation may be so great that alteration of the shape of the cells results from mechanical pressure (Edmunds). Many of the cells are in a state of fatty degeneration (Virchow).

3. *Qualitative and Quantitative Changes in the Colloid.*—The colloid is greatly diminished in amount; it may be absent. This change, however, has been also noticed in the thyroid gland of patients dying from other diseases. Some of the vesicles instead of containing colloid are filled with cells. Is this disappearance of colloid due to lessened secretion, or does it result from more active removal by the lymphatics? That is still an unsettled question.

4. *Increase in Vascularity.*—The blood vessels are distended and are increased in size; the friability of their walls has been noted and commented upon by many operators (Kummel, Kocher). This friability increases the liability to primary and to secondary hemorrhages.

5. *Changes in the Connective Tissue.*—There is an increase in the amount of connective tissue. In some cases this increase in connective tissue causes a lobulated appearance in the tumor. The fibrous septa of the gland may show some thickening at a comparatively early date. All the above mentioned histological changes may exist in small foci and not throughout the gland.

### THE CONTRASTS EXISTING BETWEEN EXOPHTHALMIC GOITRE AND MYXEDEMA.

The thyroid gland is an organ essential to the integrity of the human organism. In the absence of accessory thyroid glands, the spontaneous or gradual arrest of function of this body, or its total destruction by disease, as well as its total ablation by the surgeon, will be followed by myxedema, either acute or chronic in type. Post-operative tetany and myxedema are identical, as far as their etiology is concerned; one condition often develops into the other (Von Eiselsberg). Tetany, it would seem, is a condition of parathyroid insufficiency (Mayo, Rochester, Minn.).

The development of impending myxedema can be prevented and its manifestations controlled either by the successful transplantation of thyroid tissue in another part of the body, or by continual injections of thyroid (Vassale) or by prolonged feeding of thyroid gland (Lanz, Canter[24]).

The above facts are accepted as proofs that myxedema is a disease due to insufficiency or to absence of normally functionating thyroid tissue in the system.

The demonstration of the fact that in exophthalmic goitre we have a disease which is the diametrical opposite of myxedema in symptomatology, pathology and therapeutical indications will aid to give credence to the thyroid theory. Let us consider the evidence that contrasts the two diseases, as to the essential symptoms:

### EXOPHTHALMIC GOITRE.

1. Enlargement of the thyroid gland (almost always present).
2. Exophthalmos (a cardinal symptom).
3. Frequent presence of other ocular symptoms. Eye symptoms are of great diagnostic value, chiefly by way of information.
4. Excitable and mobile pulse, palpitation, tachycardia. Permanent tachycardia is more commonly met in exophthalmic goitre than in any other affection.
5. Exophthalmic goitre. Tremors (cardinal symptom). In 120 cases Murray noticed tremors in 111 cases.
6. Agitation, insomnia, irritability, excitability. A peculiar mental condition of nervousness is a common symptom in exophthalmic goitre.
7. More or less profuse perspiration. Skin fine, soft, moist and warm. Feel better in cold weather. Diarrhea frequent.
8. Typical myxedema may supervene on the subsidence of an equally typical exophthalmic goitre.

### MYXEDEMA.

1. Atrophy or absence of the thyroid gland (is mentioned in all the reported cases).
2. Recession of the eyeball not uncommon. In cases not consecutive to exophthalmic goitre exophthalmos is never present.
3. Absence of ocular symptoms.
4. Sluggish heart action. Bradycardia, a common symptom.
5. Myxedema. Absent, except in its rare occurrence in tetany.
6. Apathy, somnolence, dullness of apprehension and of perception.
7. Absence of perspiration even in the warmest weather. Myxedematous skin. Patients alway feel cold. Constipation common.
8. Myxedema never precedes exophthalmic goitre.

### AS TO PATHOLOGY.

### EXOPHTHALMIC GOITRE.

1. Glandular hyperplasia. Increase in number of follicles.

### MYXEDEMA.

1. Follicles are markedly diminished in number; may be absent. In case where gland is not absent there is noticed a progressive glandular atrophy.

### AS TO THERAPEUTICAL INDICATIONS.

The ingestion of thyroid preparations is almost always harmful. It aggravates the symptoms.

All measures which tend to lessen or diminish the amount of thyroid secretion are followed by improvement.

The continual ingestion of thyroid preparations is positively curative.

Implantation of gland tissue, if the latter maintains its integrity, is curative.

Our knowledge of the physiological action of thyroid gland or of its preparations is still limited. Tachycardia and increased metabolism constantly result from their ingestion. Toxic doses will cause such symptoms, as rise of temperature, insomnia, agitation, polyuria, albuminuria, complete paraplegia, etc. These symptoms we also frequently meet in cases of exophthalmic goitre. The fact that the symptom-complex of this affection can be experimentally produced by the ingestion of thyroid preparations is no longer contested. In our opinion it forms an important link in the chain of evidence supporting the thyroid theory.

Cunningham administered daily by mouth to a rabbit 1 gramme of thyroid extract; it caused exophthalmos. Edmunds[27] found that feeding dogs and monkeys large amounts of thyroid substance could bring on exophthalmos, tachycardia, loss of weight and wasting. Murray[28] obtained similar results. Notthaft[29] reports a case of a patient who took 1000 5-grain tablets of thyroid extract in five weeks. He developed all the symptoms of exophthalmic goitre; upon cessation of the drug all the symptoms promptly disappeared, with the exception of the struma and exophthalmos, which persisted for six months and then gradually disappeared.

Doyen performed a partial thyroidectomy in a case of exophthalmic goitre. Cure resulted. For some cause or other the patient took some tablets of thyroid extract, the symptoms of exophthalmic goitre recurred; with suppression of the drug the symptoms subsided. Béclerè[30] observed the development of the symptom-complex of this affection in a myxedematous woman who had taken at the beginning of the treatment 92 grammes of thyroid extract in eleven days. The drug was discontinued; the symptoms disappeared.

A critical analysis of the voluminous literature of the subject has convinced me that the following conclusions are justified:

1. Thyroid gland substance, or any of its preparations, should never be administered in the treatment of exophthalmic goitre. Their use in that disease is irrational, and it is almost invariably attended by an aggravation of the existing symptoms. Their use invariably increases the dangers of operative interference.

2. As a therapeutic agent, in the treatment of exophthalmic goitre, thymus gland substance and its various preparations are useless. Their use is, at times, attended by an aggravation of symptoms. They can not be considered curative agents.

3. Parathyroid extract as a curative agent of exophthalmic goitre has no efficacy. MacCallum says that the alterations noticed in the glandulæ parathyroidæ do not seem to be constant or sufficiently extensive to support the idea that the parathyroids have anything to do with the development of the disease known as exophthalmic goitre.

4. The medical treatment of the disease which we are now considering is, the use of belladonna being excepted, in reality largely symptomatic. For the anemia, arsenic has been given; for the nervousness and restlessness, the bromides; for the tachycardia, digitalis, strophanthus, etc. All these agents are palliative; not one has ever proven to be curative.

5. All forms of medical treatment of this affection, be they hygienic, dietetic, medicinal, organotherapic or electrical in nature, are unsatisfactory, are disappointing. Their comparative powerlessness has induced surgical endeavors to cure the disease. There is not any form of medicinal treatment which has been successful with sufficient frequency to carry conviction of its worth.

6. Serum therapy of exophthalmic goitre is as yet in an experimental state. The results attending the use of "thyroidectin" are not invariably satisfactory. Miller, Quine, Billings and others have had failures attending its employment. Their use is not devoid of dangers.

(By serum therapy is meant the employment of either (a) the serum of thyroidectomized animals, or (b) the serum of animals treated with increasing doses of thyroid extract, or (c) milk in the dried or liquid form, of thyroidectomized goats. With the use of these different sera, authors report failures and successes.)

7. It is now a demonstrated fact that all operative measures which tend to less the secretory activity of the thyroid gland, or to diminish the amount of thyroid gland tissue present in the organism, are of value in the treatment of exophthalmic goitre. That method must be chosen which at the time seems to be the least dangerous without sacrificing chances of success.

(a) Intra-glandular injections of tincture iodi, carbolic acid and other irritating liquids are unsafe in exophthalmic goitre. There is the danger of sepsis, of injecting the irritant agent into the blood vessels, of provoking alarming hemorrhage (alarming through the compression that it may exert upon the respiratory passages).

(b) The ligation of the thyroidal arteries in this disease was first recommended in 1886 by Woffler. It has been practiced by operators of such eminence as Roux, Rydigier, Kocher, etc. It is now used only as a preliminary, or as an accessory, step to partial thyroidectomy. The ligation of the four thyroid arteries is liable to determine gangrene of the thyroid gland, is liable to induce thyroid insufficiency. The objections to ligation of two or three of the thyroid arteries as a routine treatment of exophthalmic goitre are the following:

1. It is a procedure often difficult of execution, the hypertrophied thyroid gland having altered the anatomical relations of the part; the infiltration of the tissues also adds to the technical difficulties. The ligation of the vessels is especially difficult in the retro-clavicular and reto-sternal varieties of goitre.

2. Owing to the greatly increased vascularity of the organ, branches of the thyroid arteries are liable to be mistaken for the trunks of the vessels.

3. It does not secure as complete nor as permanent a mitigation of the symptoms as partial thyroidectomy, and it is, we believe, equally difficult to perform. Ligation of the inferior thyroids is just about as serious a matter as thyroidectomy. Dressman states that improvement is slower after ligature of vessels than after operative treatment on the gland.

(c) Exothyropexy for exophthalmic goitre has been performed with varying results. This operation has been termed "an unfinished partial thyroidectomy."

(d) In the absence of accessory or aberrant thyroid bodies, total thyroidectomy is very liable to be followed by cachexia strumapriva. This explains why the operation is no longer performed by those that know. Post-operative myxedema can always controlled by the administration of thyroid extract.

(e) Partial thyroidectomy is as yet the most satisfactory operation for performance in all cases of exophthalmic goitre, be they primary or secondary in type. In cases that survive the operation, it is invariably attended by marked alleviation of symptoms, in many instances by complete and permanent cure. Kocher is of the opinion that partial resection and ligature of the vessels is the most rational procedure. He first ligates the two superior thyroid arteries. This, in his opinion, is easy of execution and makes the subsequent work easier. He then ligates one inferior thyroid artery before extirpating the gland. No more thyroid tissue need be left in site than is present in the normal organism, that is from 30 to 60 grammes. The surgeons that have, for the cure of this disease, removed the largest quantity of thyroid tissue short of its entirety, are those that have obtained the very best results, both from the standpoint of the number of recoveries as well as from the standpoint of completeness of recoveries. If not enough gland tissue is removed, the maximal benefits are not derived from the operation. If too much is removed, thyroid insufficiency may develop. A small amount of glandular tissue is all that is required to maintain the ordinary nutrition of the body. When the thyroid gland is not totally removed, the possibility of post-operative myxedema can be said not to exist. Kocher met it only once in 1000 operations for goitre. In this case he removed half the gland; the remaining half atrophied. The symptoms disappeared following the administration of thyroid extract.

8. The secondary form of exophthalmic goitre, when subjected to partial thyroidectomy, almost invariably recover from the operation and from the disease.

9. Operators disagree as to the most suitable anesthesia for these cases. All the anesthetic agents have their partisans. Fatalities have occurred with all of them. Local anesthetics have the disadvantage of not completely abolishing the perception of pain. General anesthetics have the disadvantage of increasing the cardiac insufficiency, and of frequently being followed by cough which may induce secondary hemorrhage, by vomiting that may soil and infect the dressings on the wound. Kocher recommends local anesthesia; he never operates singers for goitre under general anesthesia. The Mayos (Rochester, Minn.) employ general ether anesthesia in almost all their cases. Kummell uses oxygen-chloroform. Ries uses scopalamine-morphine anesthesia. Kurt, Schulze and Riedel have seen an acute bronchitis follow operations for exophthalmic goitre in which only local anesthesia had been employed. According to Professor Fenger, the degeneration of the heart-muscle will account for some of the sudden deaths; while the absorption of thyroid, shock, anemia and general nerve exhaustion will account for most of the other deaths that are not due to the anesthetic.

10. The dangers of partial thyroidectomy in exophthalmic goitre are either avoidable, such as infection and hemorrhage, or unavoidable, such as "acute thyroidism."[32] This latter, also called "thyroid fever," is liable to occur after the observance of all precaution now known to us. We do not yet know how to prevent nor how to cure "acute thyroidism." It is not always fatal. Free drainage of the operative wound is our most serviceable weapon for combating the complication. The nature of the anesthetic, and that of the operation, seems to have little influence in its production. All Basedow patients seem very sensitive to surgical operation.

11. There is no doubt that the mortality is greater in bad cases than when the symptoms are slighter and the patient in better condition. Early operations give the best results. They give a lower percentage of deaths and a very much higher percentage of cures. Exophthalmic goitre tends to diminish vital resistance and to exhaust the nerve centers, hence operate before the patient's vitality has been lowered by chronic thyroid intoxication. Kocher lays great stress on the avoidance of the development in all cases of goitre of what he calls the "thyroid heart." This, he asserts, can be acquired either by waiting too long for surgical intervention, or by an excessive iodine or thyroid extract therapy. He assures us that the prognosis in Basedow's disease will be much better in the future, if the operation is done early.

12. Operative points:

(a) It is well to prepare patients for some time, to observe them, and to better estimate their ability to withstand operation.

(b) Place the patient in the inverted (Trendelenburg) position. Put a round pillow beneath neck so as to give better access to goitre. Maintain neck in that position which interferes least with respiration. The most rigid aseptic precautions must be observed to avoid infections: mediastinitis, deep phlegmon of neck, thrombo-phlebitis, septicemia, etc.

(c) Kocher's transverse convex incision allows of a complete exposure of both lobes. From a cosmetic standpoint, it is the best as the usual neckwear will hide the scar. If it is necessary to make a section of the sterno-hyoid and the sterno-thyroid, the Mayos advise that this be high, so as to preserve the nerve supply to these structures. Divided muscles should be sutured after extirpation of tumor. The preservation of the posterior capsule of the gland protects against many of the dangers of thyroidectomy. After completion of operation, cutaneous wound must be sutured accurately. Drain through an opening made below this wound.

(d) Hemostasis must be perfect. Do not depend on temporary compression to arrest the bleeding. It is deceptive. When possible, tie the bleeding vessels—it is preferable to leaving clamps in position. Clamps interfere with the healing of the wound. Nurses should be instructed to watch for first symptoms of secondary hemorrhage.

(e) Tissue should be left at the poles of the gland, preferably about the inferior thyroid arteries, so as to reduce the risk of injuring the recurrent laryngeal nerves.

(f) Drainage is of the utmost importance:

1. To remove what primary wound secretion is present. Although at the time of operation the bleeding may be stopped absolutely, there is always considerable oozing afterwards into the large cavity of the neck which it is impossible to obliterate by sponge pressure. This clot may cause interference with union; may cause pressure upon the trachea.

2. To remove what contents of the gland have been expressed into the wound during the operation. A certain amount of the toxic secretion of the gland being allowed to accumulate slowly in a wound that is closed will often cause such symptoms as may prove fatal in an otherwise successful case.

(g) Swab mucus away from throat. There is always a hypersecretion of mucus giving rise to troublesome coughing. This is one of the reasons why the bleeding points should be well secured for avoidance of secondary hemorrhage.

(h) Keep patient physically, mentally and emotionally quiet.

13. Recovery from all the symptoms is neither immediate nor simultaneous. The first symptom to subside is the tachycardia. The tremor and the nervous and psychical symptoms also disappear quickly. The total disappearance of menstrual disturbance is of good prognostic omen. It takes months for the entire beneficence of the operation to become manifest. The exophthalmia is the last symptom to disappear. Albert Kocher says that a total disappearance of exophthalmos can only be expected in those cases in which the operation is performed early. Eye symptoms disappear in the majority of cases quickly and completely, irrespective of persistence or disappearance of exophthalmos. The longer the period of observation after the operation, the better appear the results.

14. When, after a partial thyroidectomy, the symptoms recur, the recurrence is most frequently associated with a hypertrophy of the remaining portion of the gland. Removal of a portion of this will bring about a cure.

15. Partial thyroidectomy is indicated:

1. In all cases of secondary exophthalmic goitre.

2. In all cases of primary exophthalmic goitre.

(a) When, after three months of well-conducted appropriate medical treatment, the patient's condition is not markedly improved.

(b) When the goitre compresses or distorts the trachea, or the esophagus, or both. Long-continued dyspnea is very liable to beget pulmonary emphysema.

(c) When tachycardia is marked. Long-continued and excessive tachycardia is very liable to beget organic heart changes.

(d) When exophthalmos is so marked as to prevent complete closure of the lids during sleep. Kocher and others report cases where patients lost their eyesight through ulceration of the cornea secondary to marked exophthalmos.

(e) If the patient is losing strength.

(f) In all acute cases that seem like a sudden intoxication of the body by thyroid, even when no marked enlargement of the thyroid body can be demonstrated.

16. Surgical treatment of exophthalmic goitre is justified by theory and by facts.

BIBLIOGRAPHY.

1. Von Eiselsberg, in Von Bergman System of Practical Surgery, Phil., 1904, Vol. II, p. 357.
2. Francis P. Kinnicutt, in Loomis and Thompson American System of Practical Medicine, Vol. III, p. 693.
3. George P. Murray, Lancet, London, Vol. II, 1905, p. 1379.
4. H. H. Thompson, American Journal Medical Sciences, Phil., Dec., 1906, p. 835.
5. Dean Lewis, Surgery, Gynecology and Obstetrics, Chicago, 1906, Vol. —, p. 476.
6. Bistis, J., De l'exophthalmie unilaterale dans la maladie de Basedow. Arch. d'ophthalmologie de Paris, 1903, Vol. XXIII, p. 468.
7. Posey and Swindles, Unilateral Exophthalmos in Exophthalmic Goitre, Exophthalmic Record, Chicago, 1904, Vol. XIII, p. 204.
8. Trousseau, Clinique Ophthalmologique, Paris, 1902, April 10th.
9. W. Hale White, Med. Chir. Trans., 1885, Series 2, Vol. LXVIII, p. 221.
10. Greenfield, British Medical Journal, 1893, Vol. II, p. 1261.
11. Ehrich, Beitrage zur klin. Chirurgie, 1900, Vol. XXVIII, p. 126.
12. Temoin, Revue de Chirurgie, Vol. XVIII, 1898, p. 10.
13. Edmunds, W., Lancet, London, 1901, Vol. I, p. 1317.
14. Joffroy et Achard, Arch. de Medecine Experimentale, Paris, 1896, Vol. VI, p. 807.
15. J. J. Walsh, American Medicine, Philadelphia, 1905.
16. MacCallum, W. C., American Medicine, Philadelphia, 1905, Vol. IX, p. 934.
17. Benjamins, Ziegler's Beitrage, Vol. XXXI, 1902, April 10th.
18. Soupault Gazette Hebdomadaire de Medecine et de Chirurgie, Paris, 1897, Vol. II, p. 664.
19. Ewing, N. Y. Medical Journal, 1906, Vol. LXXXIV, p. 1061.
20. Campbell, Harry, M. D., F. R. C. P., British Med. Journal, 1902, Vol. II, p. 1420.
21. Schultze, Kurt, Mitteilung A. D. Grenzgebiete der Med. und der Chirurgie, Jena, 1906, Sechszehnter Band, p. 161.
22. MacCallum, W. C., Trans. Am. Physicians, Phil., 1905, Vol. XX, p. 352.
23. Rodocanacky, Lancet, London, 1897, Vol. II, p. 911.
24. Dr. Charles Canter, Annales de la Societe Medico-Chirurgicale de Liege, 1894, Vol. XXXII, p. 12.
25. Gifford, Ophthalmic Record, Chicago, 1906, June, Vol. XV, p. 249.
26. M. Allen Star, Loomis and Thompson, System of Practical Medicine, Vol. III, p. 693.
27. Edmunds, Walter, British Medical Journal, Oct., 1905.
28. Murray, Lancet, London, 1904, Vol. II.
29. Nothaft, Zentralblatt fuer innere Medecine, Leipzig, 1898, Vol. XIX, p. 353.
30. Beclere, Gazette des Hopituax, Paris, 1894, Vol. LXVII, p. 1114.
31. Miller, S. M., Exophthalmic Goitre, Illinois Medical Journal, Springfield, Ill., 1907, p. 253.
Billings, Frank, Exophthalmic Goitre with Report of Cases, Illinois Medical Journal, Springfield, Ill., 1907, p. 260.
Quine, Wm. E., The Medical Treatment of Exophthalmic Goitre, Illinois Medical Journal, Springfield, Ill., 1907, p. 27.
32. Barton C. Herst, Gynecological Trans., Vol. XXX, 1905.

